# Supplementary material for: AKAP6 orchestrates the nuclear envelope microtubule-organizing center by linking golgi and nucleus via AKAP9
Source: eLife. 2020 Dec 9;9:e61669. doi: 10.7554/eLife.61669 (PMC7725499; doi:10.7554/eLife.61669)
Supplement: Supplementary file 1. [file elife-61669-supp1.docx]

**Table S1**

| **Oligonucleotides** | SOURCE | IDENTIFIER |
| --- | --- | --- |
| Primers for generating pEGFP-AKAP6β by Cold Fusion   \| AKAP6β:  5-atccaccggtcgccaccatGaccaccagccaggccaaaactaaat-3 \| \| --- \| \| 5-agctcctcgcccttgctcacCCTATGCATGTTACTGTGTCGATT-3 \| \| pEGFP-N1: \| \| 5-GACACAGTAACATGCATAGGgtgagcaagggcgaggagctgttca-3 \| \| 5-tttggcctggctggtggtCatggtggcgaccggtggatcccgg-3 \| | This paper | N/A |
| Primers for silencing the siRNA site of AKAP6:   \| 5-AGTaaTgaTTtGgaCcaGgacccagagcctatgctaccc-3 \| \| --- \| \| 5-CtgGtcCaAAtcAttACTttcttcatgcacactaatgag-3 \| | This paper | N/A |
| Primers for generating pGBKT7-AKAP6-SR1 by Cold Fusion:   \| AKAP6-SR1 (585-915):  5-CCCAAGGCCGAGGTTCAGCTCTGGtcgacctgcagcggccgcataa-3 \| \| --- \| \| 5-CTGAGCCAAAGATCTGGGATCCcaggtcctcctctgagatcagct-3 \| \| pGBKT7:  5-agctgatctcagaggaggacctgGGATCCCAGATCTTTGGCTCAG-3 \| \| 5-ttatgcggccgctgcaggtcgaCCAGAGCTGAACCTCGGCCTTGGG-3 \| | This paper | N/A |
| Primers for generating pGEX4T1-AKAP6-SR1-3 by Cold Fusion   \| AKAP6-SR1-3 (585-1286):  5-ATCTGGTTCCGCGTGgatcccAgatcTTTGGCTCAGATAGCATCAA-3 \| \| --- \| \| 5-GTCACGATGCGGCCGCTCGAGtcgaCCACTTGGTAAATGTGTGGG-3  pGEX4T1: \| \| 5-GCCCACACATTTACCAAGTGGtcgaCTCGAGCGGCCGCATCGTGA-3 \| \| 5-CTATCTGAGCCAAAgatcTgggatcCACGCGGAACCAGATCCGAT-3 \| | This paper | N/A |
| Pimers for generating AKAP6-tdTomato-SR1-Farnesyl by Cold Fusion   \| mCherry-Farnesyl5:  5-TCCGGACTCAGATCTAAG-3  5-CTTGTACAGCTCGTCCATG-3  AKAP6-SR1 (585-915):  5-gcatggacgagctgtacaagTTTGGCTCAGATAGCATCAAATCC-3  5-agcttagatctgagtccggaGAGCTGAACCTCGGCCTTG-3 \| \| --- \| | This paper | N/A |
| Primers for generating AKAP6-SR1-mCherry-Farnesyl by Cold Fusion   \| AKAP6-SR1 (585-915):  5˗gtgaacgtcagatccgctagcgctaccggtcgccaccatgCAGATCTTTGGCTCAGATAGCATCAAATCCCCGG-3 \| \| --- \| \| 5˗ccttgatgatggccatgttatcctcctcgcccttgctcacccgcgatcgcCAGAGCTGAACCTCGGCCTTGGGGC-3  mCherry-Farnesyl5: \| \| 5-gcgatcgcggGTGAGCAAGGGCGAGGAGGATAACATG -3 \| \| 5-CATGGTGGCGACCGGTAGCGCTAGC-3 \| | This paper | N/A |
| Primers to generate pEGFP-AKAP6-SR1-3 (585-1286)   \| 5-gggcccAGATCTTTGGCTCAGATAGCATCAAATCC-3 \| \| --- \| \| 5-gggcccGTCGACCACTTGGTAAATGTGTGGGCTG-3 \| | This paper | N/A |
| Primers to generate pEGFP-AKAP6-SR1-2 (585-1065)  5-gggcccAGATCTTTGGCTCAGATAGCATCAAATCC-3  5-gggcccGTCGACacgtgggcccgacccactg-3 | This paper | N/A |
| Primers to generate pEGFP-AKAP6-SR1 (585-915)  5-gggcccAGATCTTTGGCTCAGATAGCATCAAATCC-3  5-gggcccGTCGACcagagctgaacctcggccttg-3 | This paper | N/A |
| Primers to generate pEGFP-AKAP6-SR2 (915-1065)   \| 5-gggcccAGATCTgccacctggaaacacagagag-3 \| \| --- \| \| 5-gggcccGTCGACacgtgggcccgacccactg-3 \| | This paper | N/A |
| Primers to generate pEGFP-AKAP6-SR3 (1065-1286)  5-gggcccAGATCTgcccacgtgacctgctatc-3  5-gggcccGTCGACCACTTGGTAAATGTGTGGGCTG-3 | This paper | N/A |
| Primers to generate mCherry-mNesprin-1α   \| gggcccgaattcATGGTGGTGGCAGAGGACTTGC gggcccGtcgaCTCAGAGTGGAGGAGGACCGTT \| \| --- \| | This paper | N/A |
| Primers to generate pCMV-Tag2b-hAKAP9-PACT   \| 5-gggcccgaattcGCCAACATTGAAGCCATCATTG-3 \| \| --- \| \| 5-gggcccgtcgacCTTCTCATGCCAGCATGAAATTG-3 \| | This paper | N/A |
| Primers to generate pCMV-Tag2b-hPcnt-PACT   \| 5-gggcccgaattcGAAAGGTCTGCTTGGAAGC-3 \| \| --- \| \| 5-gggcccgtcgacCGGGTGGCAGGATTTCTTTGAAG-3 \| | This paper | N/A |
| Primers to generate pEGFP-AKAP9-AK1b   \| 5-GGGCCCAGATCTTGATGGAAAGTGAGTTGGCTGGGAAG-3 \| \| --- \| \| 5-GGGCCCGTCGACCATTTCCTCCATCTGTGCCATGTGTTG-3 \| | This paper | N/A |
